# Epigallocatechin Gallate as a State-Dependent Modulator of Amyloid-*β*: Molecular Simulation-Guided Mechanistic Synthesis for Structure-Based Inhibitor Design

**DOI:** 10.3390/biom16050734

**Published:** 2026-05-17

**Authors:** Budimir S. Ilić

**Affiliations:** Department of Chemistry, Faculty of Medicine, University of Niš, 18000 Niš, Serbia; budimir.ilic@medfak.ni.ac.rs

**Keywords:** Amyloid-*β*, Alzheimer’s disease, (-)-epigallocatechin-3-gallate, molecular dynamics, amyloid aggregation, topology switching, *β*-sheet destabilization, interface targeting, medicinal chemistry, aggregation modulators

## Abstract

Amyloid-*β* (A*β*) aggregation is a central mechanistic feature of Alzheimer’s disease, involving heterogeneous conformational ensembles that evolve through monomeric, oligomeric, and fibrillar states. Understanding how small molecules modulate these state-dependent processes remains a major challenge in medicinal chemistry. This review examines the molecular mechanisms by which (-)-epigallocatechin-3-gallate (EGCG) perturbs A*β* aggregation, with a focus on insights derived from molecular dynamics (MD) simulations integrated with experimental data. MD studies employing structural, dynamical, and interaction-based descriptors (e.g., *β*-sheet content, contact maps, and salt bridge persistence) reveal that EGCG acts as a state-dependent modulator: it redistributes monomeric ensembles by masking aggregation-prone regions, induces topology switching in oligomers that suppresses seeding competence, and destabilizes protofibrillar *β*-sheet networks through interfacial and node-targeting interactions. Methodological analysis highlights the importance of force field selection, sampling depth, and aggregate model dependence, leading to a hierarchy of mechanistic confidence that distinguishes well-supported trends from model-specific observations. From a medicinal chemistry perspective, EGCG is best interpreted as a mechanistic probe rather than as a lead compound, informing the design of biostable modulators through principles such as bioisosteric replacement, topology control, and interfacial targeting. Collectively, this work provides a framework for translating the state-dependent aggregation mechanisms into rational therapeutic strategies.

## 1. Introduction

Alzheimer’s disease (AD) is mechanistically linked to protein misfolding and self-assembly processes that yield a spectrum of amyloid aggregates, among which the amyloid-*β* (A*β*) species occupy a central position in both mechanistic and therapeutic discussions [1,2]. In this framework, A*β* peptides populate heterogeneous conformational ensembles in solution and can progress toward oligomeric and fibrillar assemblies via complex, multi-step pathways that include nucleation, elongation, and secondary nucleation processes [2,3]. Simulation and structural studies jointly emphasize that A*β*’s intrinsic disorder enables a rapid interconversion among configurations that can seed downstream aggregation, thereby coupling monomeric ensemble properties to macroscopic assembly kinetics [4,5,6].

Multiple computational and experimental studies have emphasized that soluble A*β* oligomers are mechanistically relevant toxic entities relative to mature fibrils, motivating the strategies that remodel oligomers or perturb *β*-sheet network formation [1,5,7,8]. Consistent with this emphasis, oligomeric intermediates (dimers to higher-order assemblies) are structurally heterogeneous and kinetically dynamic in simulations, often combining fibril-like *β*-structuring with substantial disorder [9,10]. Biochemical analyses of AD brain-derived soluble fractions further implicate high molecular-weight oligomers/protofibrils as predominant soluble A*β* species, supporting a mechanistic focus on non-fibrillar assemblies [2,11].

The compound (-)-epigallocatechin-3-gallate (EGCG), a green tea catechin, interferes with amyloid assembly, including A*β*, by inhibiting fibrillization and remodeling preformed assemblies into alternative species [12,13]. Mechanistically, EGCG is notable because it remodels A*β* oligomers into off-pathway assemblies with reduced seeding competence and reduced cytotoxicity [12,13]. Its broad anti-amyloid activity across ordered/disordered protein contexts highlights the polyvalent interaction modes that are sensitive to the conformational state of the target [13,14].

Beyond its direct structural effects on A*β* aggregation, EGCG has shown biologically relevant activity in preclinical AD models. EGCG has been reported to reduce A*β* generation and cerebral amyloidosis through the promotion of non-amyloidogenic amyloid precursor protein (APP) processing, to decrease amyloid burden and tau-related pathology, to attenuate A*β*-induced neuronal injury, and to improve learning and memory readouts in cellular and animal models [15,16,17,18]. Formulation-based approaches, including EGCG-loaded nanoparticles, have further supported its biological relevance by improving brain exposure and reducing A*β* plaque burden, neuroinflammation, and cognitive deficits in APP/presenilin 1 (APP/PS1) mouse models [19]. These observations provide a physiological context for the structural remodeling mechanisms discussed in this review, while remaining preclinical, and therefore not constituting direct evidence of clinical efficacy.

Molecular dynamics (MD) simulations are well suited to EGCG–A*β* mechanistic questions because A*β* is intrinsically disordered and conformationally heterogeneous across aggregation states, while EGCG can form competing hydrogen-bonding and aromatic contacts across diverse microenvironments [4,6,13]. Enhanced sampling approaches, such as replica exchange molecular dynamics (REMD), have been important for characterizing the ensemble shifts and binding heterogeneity in A*β* systems [4,20,21].

EGCG operates as a state-dependent molecular modulator whose polyvalent hydrogen bonding and aromatic interactions redistribute A*β* conformational ensembles in a manner that differs across aggregation states: it primarily masks/reshapes monomeric hot-spot ensembles, rewires oligomer contact topology to suppress seeding competence, and destabilizes protofibril/fibril *β*-sheet networks by targeting interface and edge fragilities rather than a single binding site [12,20,22,23,24].

Rather than treating EGCG as a single-mechanism aggregation inhibitor, this review employs MD to delineate how its polyvalent interaction profile drives state-dependent ensemble redistribution across monomeric, oligomeric, and protofibrillar/fibrillar A*β* states [12,13,20,24].

This work is presented as an integrative mechanistic review rather than as a formal systematic review. Its objective is not an exhaustive enumeration of all published EGCG–A*β* molecular dynamics studies, but a critical synthesis of the most mechanistically informative computational and experimental findings to construct a unified state-dependent model of modulation. Within this framework, interface residence bias (IRB) and the MD confidence hierarchy are proposed as interpretative models that reconcile multi-pocket binding, topology switching, and protofibril destabilization within a coherent medicinal chemistry perspective. Accordingly, the manuscript should be interpreted as a mechanistic synthesis and perspective-driven review rather than as a systematic survey.

## 2. The Chemistry of EGCG

**Figure 1:**
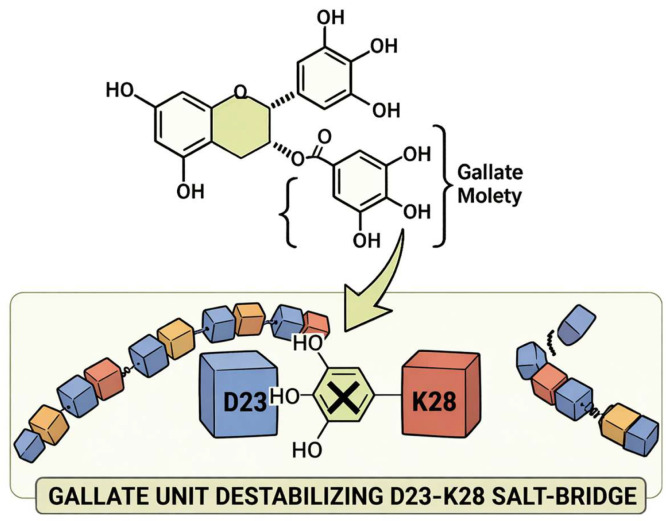
Structure–interaction determinants of EGCG relevant to A*β* modulation. The figure highlights the gallate moiety of EGCG as a key interaction element associated with perturbation of the D23–K28 salt bridge region of A*β*. This region is associated with the stabilization of protofibrillar *β*-sheet organization and provides a chemical–structural basis for the gallate-mediated disruption of aggregation-relevant A*β* interfaces.

EGCG is a catechin defined by a flavan-3-ol core esterified with a gallate moiety (Figure 1), furnishing a high density of phenolic functional groups distributed across multiple aromatic rings [13,24]. This architecture confers polyvalent interaction capability, consistent with binding modes that depend on the folding state of the protein surface [13].

Across simulation studies, EGCG’s phenolic hydroxyls form transient hydrogen bonds with polar/charged residues on A*β*, while aromatic rings engage *π*–*π*/CH–*π* interactions with aggregation-relevant side chains [23,25,26]. In protofibril simulations, the gallate ester contributes cation–*π* and hydrogen-bonding interactions (e.g., involving K28 and E11) that perturb salt bridge networks and local *β*-architecture [23,25].

EGCG’s key MD-relevant descriptors include its polyfunctionality (dense H-bond donor/acceptor capacity, multiple aromatic surfaces) and torsional adaptability (notably around the gallate ester), enabling a multi-contact engagement of fluctuating A*β* motifs [13,24]. Ensemble docking plus MD indicates that catechin-class ligands occupy multiple transient pockets on amyloid-*β* residues 1–42 [A*β*(1–42)] rather than a single rigid site [24].

EGCG is best treated as a chemical probe for interrogating *β*-sheet network fragility and aggregate plasticity, because its polyvalent interactions can reveal which A*β* contacts (salt bridge nodes, *β*-edges, grooves) are destabilizable across states [12,13,25,26]. This same binding multiplicity is mechanistically informative but limits a straightforward “single-target” lead logic, and therefore the medicinal chemistry value lies in design principles (state-biased interface engagement; topology switching; node targeting) rather than positioning EGCG itself as a lead scaffold [13,27].

## 3. The Structural Biology of Amyloid-β

Aβ monomers are intrinsically disordered yet sample structured motifs; combined MD/nuclear magnetic resonance (NMR) studies and single-molecule Förster resonance energy transfer (FRET) analyses supported by MD have shown rapidly interconverting ensembles rather than a single folded state [4,6]. REMD/NMR-constrained studies have indicated transient β-hairpins and bend motifs that expose hydrophobic residues and can promote self-association [4,20].

Assembly proceeds through intermediates with increasing conformational order and eventual in-register parallel *β*-sheet architecture [2,5]. Oligomer and protofibril landscapes are polymorphic and contain competing basins, implying that modulators can act by reshaping populations and transition propensities rather than blocking a single pathway [5,10]. Fibril-end asymmetry and end fluctuations provide a structural basis for edge vulnerabilities relevant to interface-acting ligands [22,28].

Within this review, protofibrils are defined as metastable, partially ordered assembly intermediates that possess substantial *β*-sheet organization but retain greater structural plasticity, incomplete registry, and exposed interfacial vulnerabilities relative to mature fibrils [29,30]. By contrast, mature fibrils are defined as more persistent, highly ordered assemblies characterized by extended in-register parallel *β*-sheet architecture, greater longitudinal order, and higher structural stability [30,31,32]. This distinction is mechanistically important because EGCG primarily exploits the dynamic interfaces and node fragilities of protofibrillar assemblies, whereas mature fibrils present more rigid and less accessible interaction landscapes [23,25].

Residues Asp23 (D23) and Lys28 (K28) define a key electrostatic and bend-forming region within A*β* assemblies [33,34]. The D23–K28 salt bridge has been widely implicated in the stabilization of the central turn/bend region, promotion of aggregation-prone conformational states, and maintenance of *β*-structured oligomeric or protofibrillar architectures [33,35]. In A*β*(1–42) protofibrillar models, the D23/K28 region contributes to *β*-sheet organization and protofibril stability, although the exact stabilizing contacts involving K28 may vary depending on the A*β* polymorph or structural model considered [35,36,37]. Molecular dynamics studies further suggest that EGCG can destabilize this structurally sensitive region by reducing the *β*-sheet content, disrupting hydrogen-bond networks, and perturbing K28-associated salt bridge interactions [23,25]. In particular, the gallate moiety of EGCG has been proposed to support cation–*π* interactions with K28, together with hydrogen-bonding contacts involving nearby polar or terminal groups, thereby contributing to local interfacial destabilization and protofibril remodeling [23,25].

In line with this polymorphic landscape, A*β* monomers and aggregates expose dynamic hydrophobic patches and interfacial grooves that can act as ligandable motifs; inhibitors can destabilize assemblies by perturbing these networks rather than solely capping fibril ends [25,26,38,39]. Because motifs are state-dependent, mechanisms inferred from monomers cannot be reliably extrapolated to protofibrils/fibrils [20,23,25,26].

## 4. Molecular Dynamics Insights into EGCG–Aβ Interactions

**Figure 2:**
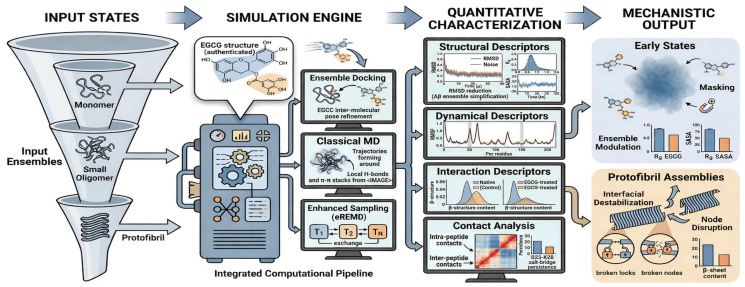
Computational workflow for the state-resolved analysis of EGCG–A*β* interactions. State-dependent EGCG–A*β* mechanisms are resolved through an integrated computational pipeline combining ensemble docking, classical molecular dynamics, and enhanced sampling (e.g., REMD). This framework enables the quantitative characterization of ligand-induced effects using structural (RMSD, Rg, SASA), dynamical (RMSF), and interaction-based descriptors, including *β*-structure content, salt bridge persistence, and contact map redistribution. Together, these analyses distinguish ensemble modulation in early states from interfacial destabilization in protofibrillar assemblies.

**Figure 3:**
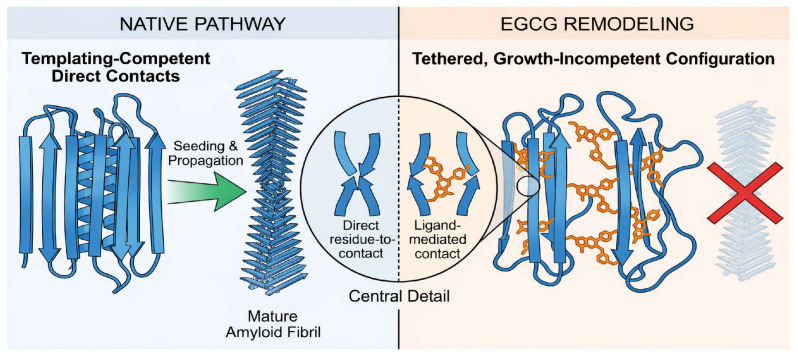
EGCG-induced redistribution of A*β* contact networks across aggregation states. EGCG remodels A*β* interaction networks by reducing the *β*-region contact density and redistributing the intra- and inter-peptide interactions across aggregation states. In oligomeric assemblies, this manifests as a shift from templating-competent direct contacts to tethered, growth-incompetent configurations, consistent with experimentally observed suppression of seeding. These contact network changes provide a structural basis for EGCG-driven ensemble redistribution and inhibition of aggregation propagation.

Molecular dynamics simulations provide a quantitative, atomistic framework for resolving how EGCG modulates A*β* conformational ensembles and interfacial interactions across distinct aggregation states (Figure 2). EGCG–A*β* molecular dynamics simulations have characterized ligand-induced effects using a combination of structural descriptors, including root mean square deviation (RMSD), radius of gyration (Rg), and solvent-accessible surface area (SASA), as well as dynamical descriptors such as root mean square fluctuation (RMSF) and interaction-based descriptors, including *β*-structure content, interpeptide hydrogen-bond networks, salt bridge persistence, and ligand–peptide contact maps (Figure 3) [23,25,26,40,41]. Collectively, these metrics resolve whether EGCG primarily drives ensemble redistribution in monomeric/dimeric states or disrupts interfacial *β*-sheet networks in protofibrillar assemblies [23,40]. These descriptors collectively define the structural and dynamical signatures of EGCG-induced modulation across aggregation states (Table 1).

EGCG forms multivalent interaction networks with A*β* combining hydrogen bonding and aromatic contacts [23,25,26]. These interaction modes can be systematically mapped to their state-dependent mechanistic roles, as summarized in Table 2. Protofibril simulations identify the disruption of stabilizing contacts and increased disorder at interfaces, consistent with competitive interference with *β*-sheet hydrogen bonding and hydrophobic packing [23,25]. Oligomer-level studies similarly point to redistributed interpeptide contacts and reduced *β*-organization [12,40].

**Figure 4:**
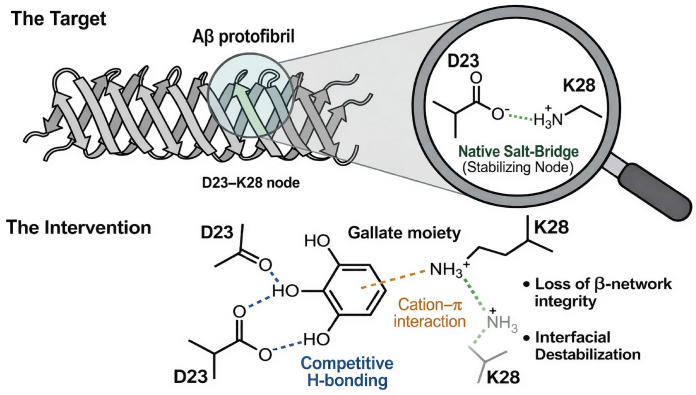
Mechanistic disruption of protofibril salt bridge networks by EGCG. EGCG destabilizes the protofibril architecture by targeting interfacial stabilizing interactions, particularly salt bridge networks centered on residues such as D23–K28. Gallate-mediated cation–*π* interactions, combined with multivalent hydrogen bonding, weaken the electrostatic and hydrogen-bonding networks that maintain *β*-sheet alignment. This disruption of interfacial nodes leads to loss of *β*-network integrity and underlies the enhanced protofibril-disruptive capacity of EGCG relative to non-gallated catechins.

REMD simulations of A*β*(1–42) dimers have shown that EGCG expands dimer conformations, reduces interchain contacts and *β*-sheet content, and reshapes free energy basins toward less fibril-prone states [40]. Protofibril simulations have shown that EGCG destabilizes LS-shaped A*β*(1–42) protofibrils via gallate-enabled cation–*π* interactions (notably with K28, Figure 4) and hydrogen bonding that perturbs salt bridges and *β*-architecture [23]. Experimentally constrained NMR data, including dark-state exchange saturation transfer (DEST), have demonstrated that EGCG remodels amyloid-*β* residues 1–40 [A*β*(1–40)] oligomers into seeding-incompetent assemblies by shifting the monomer–protofibril contact topology from direct to tethered contacts without releasing the monomers [12].

Topology switching refers to the conversion of productive, templating *β*-region interpeptide contacts (‘direct contacts’) into non-productive, growth-incompetent tethered configurations (‘tethered contacts’), in which the monomers remain associated with oligomeric assemblies but lose the geometric alignment required for fibril elongation and secondary nucleation [12]. Operationally, this process is reflected by redistribution in contact maps, reduced persistence of aligned *β*-strand interactions, and preservation of association without monomer release [12]. Thus, inhibition arises from altered contact topology rather than aggregate dissociation.

Enhanced sampling (REMD; metadynamics in related contexts) is essential because A*β* landscapes are rugged and disordered ensembles are sampling-sensitive [4,20,43]. Coarse-grained landscape work has shown multiple metastable oligomer basins and conversion kinetic barriers, supporting a mechanistic view in which modulators act by redistributing populations and barriers [5]. EGCG effects in cross-amyloid contexts involving A*β* and human islet amyloid polypeptide (hIAPP) include diminished *β*-sheet structure and weakened inter- and intrachain interactions through hydrogen bonding, *π*–*π* stacking, and cation–*π* interactions, consistent with an ensemble-redistribution mechanism [43,44,45].

Monomeric ensemble docking/MD indicates that catechins occupy multiple pockets and can block the central hydrophobic region, implying early-stage hot-spot masking rather than a unique bound state [24]. Oligomer studies have shown reshaping of basins and contact dilution [40], while oligomer remodeling experiments have constrained the functional mechanism as contact–topology switching to suppress seeding [12]. Protofibril studies have emphasized the disruption of salt bridges and *β*-network integrity at interfaces, with the gallate ester group of EGCG imparting enhanced disruptive capacity relative to epigallocatechin (EGC) [23,25].

## 5. Mechanistic Interpretation

### 5.1. State-Dependent Mechanistic Model of EGCG–Aβ Modulation

**Figure 5:**
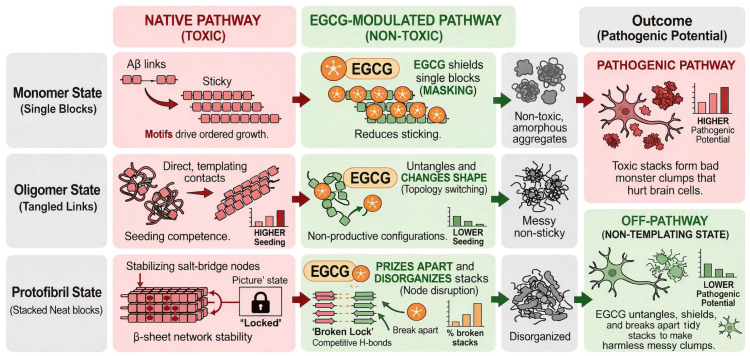
Unified state-dependent mechanism of EGCG modulation across A*β* aggregation pathways. EGCG modulates A*β* aggregation through a hierarchy of state-dependent mechanisms defined by its polyvalent interaction profile. In monomeric ensembles, EGCG drives the redistribution of conformational populations and masks aggregation-prone motifs. In oligomeric states, topology switching from direct to tethered *β*-region contacts is experimentally constrained by NMR/DEST data, whereas current MD simulations provide compatible network-level support through reduced *β*-sheet content and weakened interchain contacts rather than through direct trajectory-resolved reproduction of the full geometric transition. In protofibrillar assemblies, EGCG preferentially targets interfacial nodes, disrupting salt bridges and weakening *β*-sheet networks. These coordinated effects shift A*β* toward off-pathway, non-templating states with reduced structural stability and pathogenic potential.

EGCG’s mechanism is most coherently described as a state-dependent network perturbation in which its effects vary across aggregation states (Figure 5). Building on the IRB concept introduced in the Introduction, EGCG’s mechanistic effect can be understood as preferential and persistent residence at aggregation-relevant interfaces, including *β*-sheet edges, protofibril grooves, and salt bridge nodes, where transient binding events may exert disproportionate effects on assembly stability. Unlike classical affinity-driven models centered on a dominant binding site, IRB emphasizes state-dependent residence at destabilizable network nodes. In monomeric ensembles, this bias is weak and distributed across multiple transient pockets [20,24]; in oligomers, it manifests through localized topology switching within *β*-regions [12]; and in protofibrils, it becomes strongest through the preferential engagement of stabilizing interfacial nodes, such as the D23–K28 region [23,25]. Thus, IRB provides a unifying framework linking multi-pocket binding, topology switching, and protofibril destabilization within a single state-resolved mechanistic model.

Consistent with this framework, EGCG engages multiple transient binding pockets in monomeric ensembles and masks aggregation-prone motifs, resulting in ensemble redistribution rather than formation of a stable 1:1 complex [20,24]. In oligomeric assemblies, EGCG reduces the *β*-structure and interchain contact density, but NMR/DEST experiments further constrain its functional role as enforcing a switch in *β*-region engagement from direct to tethered interactions, thereby suppressing seeding without monomer release [12,40]. In protofibrils and fibrils, EGCG preferentially targets interfacial stabilizers, including salt bridges, key hydrogen bonds, and groove/edge contacts, where gallate-mediated cation–*π* and hydrogen-bonding interactions promote destabilization of the *β*-network architecture [22,23,25].

The direct-tethered contact transition should be interpreted as an experimentally constrained topology switch defined by NMR/DEST measurements rather than as a fully reproduced MD-derived geometric transition [12,46]. Current MD simulations support this mechanism indirectly by showing reduced *β*-sheet content, weakened interchain contacts, and redistribution of contact networks toward less aggregation-prone conformational states [40]. However, they do not yet provide a direct contact distance distribution or trajectory-resolved geometric conversion from direct to tethered *β*-region engagement. Accordingly, MD should be viewed as providing atomistic context for the experimentally observed suppression of seeding competence, while future simulations should explicitly quantify interpeptide contact distances, *β*-region contact maps, and topology-specific order parameters capable of distinguishing direct from tethered configurations.

Experimentally resolved remodeling mechanisms further indicate that EGCG suppresses seeding competence by changing the monomer–protofibril contact topology in *β*-regions (direct-tethered), preventing productive templating interactions without releasing the monomers [12]. This aligns with MD observations that EGCG reduces *β*-sheet organization and interchain connectivity in early oligomers, thereby reducing the probability of forming a templating-competent architecture [40], and with protofibril simulations showing the destabilization of interfacial stabilizers (salt bridges and key H-bonds) [23,25].

EGCG “selectivity” is best understood as state bias, including multi-pocket engagement in monomer ensembles [24], topology-switching remodeling in oligomers [12], and node/edge destabilization in protofibrils [23,25]. This state conditionality reinforces a unifying interpretation in which EGCG’s apparent selectivity emerges from conformational-state dependence rather than intrinsic binding specificity, consistent with its experimentally supported behavior across ordered and disordered protein targets [13].

### 5.2. Methodological Limitations in MD Studies of Aβ: Force Field, Sampling, and Model Dependence

A rigorous interpretation of the MD mechanisms in A*β* systems requires an explicit recognition of methodology-dependent variability, which is particularly pronounced due to the intrinsic disorder, polymorphism, and multi-scale aggregation behavior of A*β* assemblies [32,33]. Consequently, the confidence and transferability of mechanistic conclusions drawn from EGCG–A*β* simulations are constrained by the following three interdependent factors: force field dependence, sampling limitations, and aggregate model dependence.

#### 5.2.1. Force Field Dependence

Biomolecular force fields differ in their parameterization of backbone conformational energetics and non-bonded interactions, leading to substantial variability in predicted secondary-structure propensities and intermolecular contact patterns in intrinsically disordered peptides such as A*β* [47,48].

Representative force fields commonly employed in A*β* simulations include AMBER ff99SB/ff14SB, CHARMM36m, and OPLS-AA/M, which differ in backbone torsional balance, side-chain packing, water model coupling, and non-bonded interaction parametrization [20,48,49,50]. Comparative studies have shown that AMBER, CHARMM, OPLS, and related force field families can yield different secondary-structure propensities, interpeptide contact patterns, and aggregation kinetics in A*β* systems [48,51,52]. These Hamiltonian-dependent variations directly influence the predicted *β*-sheet content, salt bridge persistence, and residue-level hotspot identification, reinforcing that a mechanistic interpretation should prioritize reproducible ensemble-level trends over single-site narratives.

Similarly, detailed analyses of A*β*(1–42) monomer ensembles reveal that different force fields yield distinct conformational distributions and intramolecular interaction patterns, directly impacting the inferred ligand-binding landscapes [53]. These observations imply that the residue-level interaction “hotspots” and binding site preferences identified in EGCG–A*β* simulations are inherently Hamiltonian-conditional rather than universally transferable. Accordingly, a well-supported mechanistic interpretation should prioritize ensemble-level observables, such as global *β*-structure content, contact map redistribution, and network-level perturbations, over single-residue binding narratives, and, where possible, should be validated across multiple force fields [47,48].

#### 5.2.2. Sampling Limitations

Sampling remains a dominant source of uncertainty in the simulations of disordered and aggregating peptide systems, as the underlying free energy landscapes are rugged and characterized by high kinetic barriers separating metastable states [22,54]. Conventional MD trajectories are often insufficient to capture rare but mechanistically critical events, such as interfacial detachment, salt bridge disruption, or oligomer restructuring. Enhanced-sampling approaches, including REMD, replica exchange with solute tempering 2 (REST2), metadynamics, and related free energy techniques, are therefore essential for achieving a statistically meaningful exploration of the conformational space [22,54]. In large A*β* assemblies, microsecond-scale simulations combined with replica-based methods have been required to resolve slow structural rearrangements and fragmentation pathways, highlighting the dependence of mechanistic conclusions on sampling depth and strategy [54]. Consequently, interpretations derived from limited sampling should be regarded as provisional unless supported by convergence analysis, independent replicates, or enhanced-sampling frameworks specifically designed to overcome known kinetic barriers.

#### 5.2.3. Aggregate Model Dependence

Mechanistic conclusions drawn at the aggregate level are strongly conditioned by the structural model employed, including polymorph selection, oligomer size, protofibril architecture, and interface definition.

To improve structural transparency, the representative structural model sources used in EGCG–A*β* simulation studies are summarized in Supplementary Table S1. These include REMD/NMR-derived monomer ensembles, EGCG-remodeled oligomeric states constrained by NMR/DEST experiments, and LS-shaped A*β*(1–42) protofibril/fibril models derived from cryo-EM structural studies [4,12,20,23,25,30,31,40,55,56,57]. Because these models differ in interface accessibility, packing geometry, fibril-end architecture, and exposed salt bridge or groove regions, mechanistic conclusions involving node targeting, D23–K28/K28-associated disruption, and edge destabilization should be interpreted as partially model-dependent.

Amyloid fibrils are intrinsically polymorphic, with multiple experimentally observed structural variants differing in packing, registry, and interfacial organization [58]. Solid-state NMR and morphometric analyses demonstrate that distinct fibril polymorphs coexist and exhibit unique structural features, implying that any single structural model represents only a subset of the accessible aggregation landscape [58]. MD studies have further shown that protofibril orientation, lateral association, and interface topology can vary substantially, leading to different disruption pathways and ligand interaction patterns [54,59]. As a result, mechanistic insights, particularly those involving interfacial destabilization or edge targeting, should be interpreted as either consistent across structural models or conditional on specific polymorphic contexts. A generalization of model-dependent findings therefore requires validation across multiple aggregate architectures and construct definitions.

Taken together, these methodological considerations establish that a reliable mechanistic interpretation of EGCG–A*β* simulations depends on distinguishing ensemble-level, well-supported trends (e.g., global *β*-sheet reduction, contact network redistribution, interfacial destabilization) from model-dependent features (e.g., residue-specific binding sites or polymorph-specific edge effects). This distinction provides the conceptual basis for the classification framework introduced in Table 3, which is derived from a qualitative cross-study comparison of reproducibility across force fields, sensitivity to sampling protocols, and consistency across aggregate models. Accordingly, mechanistic claims in EGCG–A*β* systems should be interpreted within a hierarchy of confidence that explicitly reflects force field, sampling, and model dependencies, rather than as universally transferable molecular mechanisms.

The mechanistic confidence levels in Table 3 were assigned using a qualitative cross-study assessment based on reproducibility across force fields, sensitivity to sampling depth or enhanced sampling, dependence on aggregate model or polymorph choice, and the presence of independent experimental constraints [48,52,60,61]. Observations reproduced across multiple simulation settings and supported by independent experimental constraints, such as NMR/DEST data, were classified as high or very high confidence [12]. By contrast, residue-specific binding claims or polymorph-restricted effects were classified as medium or lower confidence when strongly dependent on force field choice, limited sampling or specific aggregate constructs.

#### 5.2.4. MD Confidence Hierarchy

The MD confidence hierarchy introduced in this review provides a structured framework for interpreting mechanistic claims in EGCG–A*β* systems according to consistency across simulation conditions and experimental validation. At the highest level are experimentally constrained mechanisms, such as topology switching defined by DEST/NMR data [12]. Intermediate confidence includes reproducible ensemble-level observations, such as *β*-sheet reduction, contact map redistribution, and interfacial destabilization, particularly when supported across multiple simulation settings or aggregate models [23,25,40]. Lower confidence applies to residue-specific dominant binding sites or polymorph-restricted edge effects that remain strongly Hamiltonian-, sampling-, and model-dependent [48,52,60,61]. This hierarchy is intended to prevent the overinterpretation of simulation-specific observations and to prioritize mechanistically transferable conclusions.

To improve interpretability, mechanistic conclusions are explicitly classified into the following three categories: experimentally constrained mechanisms, such as topology switching defined by DEST/NMR data [12]; high-confidence MD-supported observations, such as *β*-sheet reduction, contact map redistribution, and interfacial destabilization reproduced across multiple simulation conditions [23,25,40]; and model-dependent MD-informed hypotheses, such as residue-specific dominant binding sites or polymorph-restricted edge effects that remain sensitive to force field selection, sampling depth, and aggregate model choice [48,52,60,61]. This structure preserves mechanistic rigor while avoiding artificial quantitative precision in cases where a direct cross-study numerical comparison is not methodologically justified.

**Table 3:** Evidence Categories and Mechanistic Confidence Levels in EGCG–A*β* Studies. Mechanistic conclusions are classified as experimentally constrained mechanisms, high-confidence MD-supported observations, or model-dependent MD-informed hypotheses. Confidence levels were assigned qualitatively based on experimental support, cross-study reproducibility, force field sensitivity, sampling depth, and aggregate model dependence.

Observation	Evidence Category	Reproducibility Across Force Fields	Sensitivity to Sampling Length	Aggregate Model Dependence	Mechanistic Confidence Level
EGCG reduces *β*-sheet content and interchain contacts in early oligomers (e.g., dimers)	High-confidence MD-supported observation	Moderate (directional trend consistent; monomer ensembles vary) [20,40]	High (needs enhanced sampling to stabilize ensemble statistics) [4,40]	Moderate (depends on oligomer construct) [40,62]	High (trend-level)
Oligomer remodeling suppresses seeding via direct-tethered topology switching without monomer release	Experimentally constrained mechanism	N/A (experiment-defined) [12]	Low (not a simulation artefact) [12]	Moderate (shown for A*β*(1–40); extension to A*β*(1–42) requires care) [2,12]	Very high
Protofibril disruption involves salt bridge node weakening and interface destabilization (K28-associated; D23–K28)	MD-supported, model-conditional observation	Moderate (salt bridges sensitive to solvent/ion models) [20,23,25]	Moderate–High (rare rupture events) [23,25]	High (polymorph choice matters, e.g., LS-shaped protofibril) [23]	Medium–High (model-conditional)
“Single dominant binding site” for EGCG on monomeric A*β*	Model-dependent MD-informed hypothesis/potential overinterpretation	Low (multi-pocket ensemble binding is typical) [20,24]	High (pocket ranking shifts with sampling) [4,24]	Moderate (A*β*(1–40) vs. A*β*(1–42); construct differences) [4,6]	Low (potentially artefactual if overinterpreted)
Edge/end targeting as primary mode (via fibril-end asymmetry)	Model-dependent MD-informed hypothesis	Indirect (end asymmetry consistently observed; EGCG-specific tests limited) [22]	Moderate (end opening is fluctuation-driven) [22]	High (end construction, polymorph truncation) [22,28]	Medium (plausible, incompletely resolved)

### 5.3. Medicinal Chemistry Perspective: Derivatives, Stability, Metabolism, and Structure–Activity Relationship (SAR) Implications

Although EGCG exhibits limited systemic bioavailability and undergoes rapid auto-oxidative and metabolic transformation, its polyphenolic scaffold and gallate moiety define the interaction motifs highly relevant to A*β* aggregation modulation [63,64,65].

EGCG undergoes spontaneous oxidation to quinone-like products, which modifies its hydrogen-bonding profile and may alter aggregation pathways [65]. Additionally, rapid phase II metabolism, including extensive glucuronidation, sulfation, and O-methylation, substantially reduces the systemic levels of the active parent compound in vivo [64,66,67]. These liabilities highlight the need for chemically stabilized analogues or scaffold redesign, as evidenced by the chemical instability of EGCG and ongoing efforts in structural modification to enhance stability and bioavailability [68,69,70]. These physicochemical and metabolic liabilities explain why EGCG is best viewed as a mechanistic template rather than as a direct therapeutic lead; scaffold-hopping strategies for transferring these features into a drug-like chemical space are discussed separately in Section 5.8.

Gallate-free catechins, such as EGC, exhibit markedly reduced cation–*π* engagement and a diminished protofibril remodeling capacity, underscoring the mechanistic primacy of the gallate moiety in driving the EGCG–A*β* interactions [65,71,72]. Epimerized gallate-containing derivatives, such as gallocatechin gallate (GCG) and catechin gallate (CG), exhibit altered torsional flexibility and steric profiles, leading to distinct conformational ensembles and engagement modes [63,73,74]. These observations define a preliminary structure–activity landscape for catechin-like modulators and provide a mechanistic basis for rational scaffold optimization [65,71,72].

### 5.4. Integration with Experimental Data

A representative example of experiment–simulation convergence is provided by the NMR/DEST study of Ahmed et al., in which EGCG remodeled A*β*(1–40) oligomers into seeding-incompetent assemblies by shifting the *β*-region interactions from direct templating contacts to tethered configurations without monomer release [12]. Complementary REMD simulations of A*β*(1–42) dimers showed reduced interchain and intrachain contacts, lower *β*-sheet content, and redistribution of free energy basins toward less fibril-prone conformations in the presence of EGCG [40]. Together, these findings indicate that MD provides an atomistic context for experimentally observed suppression of seeding competence, while not independently proving the full direct-tethered transition.

Immuno-infrared sensor measurements combined with extended MD and ab initio calculations further support EGCG-induced fibril degradation through the rupture of interchain hydrogen bonds, consistent with the *β*-network destabilization inferred from protofibril simulations [41,42]. Thus, the strongest experiment–simulation convergence occurs at the network level, including *β*-sheet loss, weakened interchain connectivity, and destabilization of the stabilizing nodes, whereas residue-specific binding-site assignments remain more model-sensitive [20,23,24,41].

### 5.5. Limitations of Current MD Research

Force field dependence in A*β* monomer ensembles constrains the residue-specific binding claims and demands an ensemble-level interpretation [6,20]. Aggregate models (protofibril polymorph choice; finite-size; end effects) influence the observed disruption pathways, especially when edge asymmetry and open/closed end dynamics differ [22,23]. Sampling limitations remain central because rare events (such as salt bridge rupture or interface detachment) may be underrepresented without enhanced sampling [4,40,43]. Therefore, mechanistic conclusions require explicit classification into well-supported versus model-dependent categories (Table 3) [12,20,23]. Accordingly, mechanistic claims in EGCG–A*β* systems should be interpreted within a hierarchy of confidence that explicitly reflects force field, sampling, and model dependencies, rather than as universally transferable molecular mechanisms.

### 5.6. Polymorph- and Condition-Dependent Limits of EGCG Remodeling

Direct evidence that EGCG promotes the aggregation of specific structurally defined A*β* polymorphs remains limited. However, several studies have indicated that EGCG activity is not universally invariant and may depend on aggregate state, fibril architecture, concentration, oxidation state, and assay environment. For example, EGCG can remodel mature A*β* fibrils and reduce cellular toxicity in some experimental settings [72], whereas kinetic studies of A*β*42 aggregation have reported concentration-dependent bimodal effects, with high EGCG to A*β*42 ratios accelerating the aggregation rate despite reducing the final fibril yield [75]. In addition, artificial cerebrospinal fluid conditions can markedly alter the inhibitor performance and render oxidized EGCG ineffective in A*β* aggregation assays [76]. These observations suggest that apparent EGCG efficacy should be interpreted as condition- and state-dependent rather than universally applicable across all A*β* assemblies or polymorphic contexts. Future studies should explicitly compare EGCG effects across structurally defined A*β* polymorphs and maturation states to distinguish general anti-amyloid remodeling from polymorph-selective resistance or assay-dependent behavior.

### 5.7. Peracetylated Derivatives and Prodrug Strategies for Enhanced CNS Penetration

The therapeutic translation of EGCG is fundamentally obstructed by its poor pharmacokinetic profile, primarily defined by high susceptibility to auto-oxidation at physiological pH and rapid sequestration through Phase II metabolic pathways [63,70]. To circumvent these systemic barriers, medicinal chemistry efforts have focused on prodrug strategies, particularly the development of peracetylated EGCG (Pro-EGCG/AcEGCG) and various lipophilic fatty acid esters [69,70]. By transiently masking the eight phenolic hydroxyl groups with acetyl or acyl moieties, these derivatives can shield the molecule from premature degradation while increasing lipophilicity, a property relevant to membrane permeability and potentially improved CNS-oriented delivery [68,70].

Once within the target cells or relevant tissue compartments, these masked phenolic groups may be liberated via endogenous esterases, regenerating the active polyphenol in situ [70]. Previous studies on Pro-EGCG/AcEGCG have demonstrated improved cellular uptake, intracellular conversion to EGCG, and increased in vivo bioavailability relative to the parent compound, supporting peracetylation as a strategy for protecting EGCG from rapid degradation and metabolic inactivation prior to intracellular release [77]. Similarly, lipophilic acyl derivatives, such as 4′-O-palmitoyl EGCG, exhibit greater chemical stability than native EGCG, supporting acyl/prodrug masking strategies as a route for improving pharmacokinetic behavior and sustained biological activity [78,79].

Comprehensive SAR analyses and MD simulations have suggested that, while fully protected scaffolds may temporarily lose the hydrogen-bond donor capacity necessary for direct A*β* network disruption, controlled release of the parent EGCG may sustain exposure to the bioactive species at the relevant nodes of protein misfolding [12,23]. Thus, peracetylated and acylated EGCG derivatives should be viewed primarily as pharmacokinetic optimization strategies rather than as direct substitutes for the interaction profile of unmodified EGCG.

### 5.8. Scaffold Hopping: From Catechin Polyphenols to Biostable Amyloid Modulators

The medicinal chemistry landscape of A*β* modulation is increasingly shifting from natural polyphenols toward chemically stable, biostable mimetics that preserve key mechanistic features of aggregation-disrupting ligands. While EGCG provides a mechanistically informative template, its intrinsic limitations, including redox instability, rapid phase II metabolism, and potential classification as a pan-assay interference compound (PAINS), restrict its direct therapeutic applicability [63,70].

Scaffold hopping represents a rational strategy to overcome these liabilities by retaining the essential pharmacophoric elements responsible for A*β* modulation, namely as follows: (i) aromatic surfaces enabling *π*–*π* and CH–*π* interactions; (ii) a high electron-density motif capable of cation–*π* engagement, functionally analogous to the gallate group; and (iii) sufficient conformational adaptability to engage dynamic, state-dependent binding environments. Experimental evidence has demonstrated that EGCG remodels amyloidogenic polypeptides into off-pathway, non-templating oligomers with reduced cytotoxicity, while NMR/DEST studies have shown that EGCG suppresses seeding competence by shifting the *β*-region contact topology from direct to tethered interactions [12,71]. This experimentally validated mechanism provides a functional benchmark for evaluating next-generation scaffolds.

Building on the SAR limitations outlined in Section 5.3, scaffold-hopping strategies should prioritize chemically stable aromatic or heterocyclic frameworks capable of preserving the *π*–*π*, CH–*π*, and cation–*π* interaction potential while reducing the metabolic liabilities of the catechin/gallate architecture [65,71,72,80]. Nitrogen-containing heterocycles, including quinoline- and isoquinoline-based scaffolds, as well as other aromatic frameworks identified through structure- and shape-based screening, may reproduce EGCG-like interaction geometries while offering improved metabolic stability and blood–brain barrier (BBB) permeability [24,81]. In parallel, lipophilicity should be tuned to enhance access to hydrophobic interfacial grooves on amyloid surfaces while avoiding non-specific hydrophobic collapse [82], and conformational constraint may help orient interaction vectors toward destabilizable *β*-network nodes [83,84].

Importantly, the objective of scaffold hopping in this context is not to reproduce a single binding mode, but to preserve the mechanistic function of EGCG, including the state-dependent modulation of A*β* assemblies through contact network redistribution, topology switching, and destabilization of *β*-sheet stabilizing interactions. Accordingly, scaffold hopping should be viewed as a strategy for translating EGCG’s mechanistic signature into a drug-like chemical space, enabling the development of small molecules that retain their aggregation-modulating activity while meeting the physicochemical and pharmacokinetic requirements necessary for central nervous system therapeutics.

### 5.9. PAINS Liability, Assay Artefacts, and Orthogonal Validation

Although EGCG is mechanistically informative, its polyphenolic and redox-active structure introduces PAINS-like liabilities that can complicate the interpretation of Aβ aggregation assays [85,86]. In particular, ThT fluorescence inhibition may reflect true β-sheet reduction, but may also arise from fluorescence quenching, competitive dye displacement, optical interference, or compound–fibril interactions unrelated to genuine remodeling [87,88,89]. Therefore, EGCG and future derivatives should be evaluated using orthogonal workflows that combine dye-independent kinetic assays, NMR/DEST–NMR, ITC, CD/FTIR spectroscopy, electron microscopy with appropriate controls, and cell-based toxicity or seeding assays. From a design perspective, next-generation derivatives should preserve productive aromatic and hydrogen-bonding interactions while reducing redox cycling, catechol/quinone reactivity, non-specific protein binding, and assay-interfering optical properties.

### 5.10. Translational Gap

Despite strong mechanistic evidence that EGCG can remodel A*β* assemblies in vitro and in simulation-derived models, its therapeutic translation remains limited by poor systemic bioavailability, rapid metabolism, chemical instability, and insufficiently characterized exposure within the cerebrospinal fluid and brain parenchyma [64,90]. AD animal studies and formulation-based approaches have suggested that EGCG or EGCG-loaded nanoparticles can reduce amyloid burden, attenuate neuroinflammation, and improve cognitive readouts; however, these effects are formulation-, dose-, and model-dependent [18,19,91,92]. For example, EGCG/ascorbic acid-loaded PEGylated PLGA nanoparticles increased long-term plasma and brain EGCG exposure relative to free EGCG and improved amyloid, inflammatory, synaptic, and behavioral endpoints in APP/PS1 mice [19]. Nevertheless, current studies have yet to establish a direct PK/PD relationship between free EGCG or active derivatives in cerebrospinal fluid (CSF) and brain parenchyma and specific A*β*-remodeling mechanisms. Future translational studies should therefore quantify plasma, CSF, and brain parenchymal exposure of EGCG, Pro-EGCG, and lipophilic/acylated derivatives, while linking these concentrations to target engagement, aggregate remodeling, seeding suppression, and behavioral outcomes in AD-relevant models. This gap underscores why EGCG should be considered a mechanistic probe and scaffold template rather than a validated therapeutic lead.

## 6. Therapeutically Oriented Conclusions and Design Principles

To align this review with medicinal chemistry expectations, the conclusion must transition from descriptive mechanistic insights to actionable therapeutic principles. Effective modulators of A*β* aggregation should exhibit state-dependent engagement, interacting differentially with monomers, oligomers, and protofibrils in a manner that mirrors EGCG’s polyvalent, context-dependent profile. A central mechanistic requirement is the enforcement of topology switching, in which small molecules stabilize the tethered, non-templating *β*-region contacts that suppress seeding without releasing monomers. In addition, therapeutically useful scaffolds should selectively destabilize *β*-network nodes, most notably the D23–K28 salt bridge region and *β*-edge vulnerabilities, which represent intrinsic structural weak points within protofibrils. Molecular polyvalency must be optimized such that aromatic surfaces and hydrogen-bonding elements are preserved to maintain their disruptive capacity while avoiding excessive binding multiplicity. Finally, unlike EGCG, next-generation modulators must incorporate chemically and metabolically stable motifs with improved BBB permeability while retaining the essential cation–*π* and hydrogen-bonding geometries that govern mechanistic efficacy. Based on these mechanistic and medicinal chemistry considerations, the following integrated design principles can be proposed.

The feasibility of translating EGCG from a mechanistic probe into a design template is supported by broader amyloid-inhibitor SAR precedents. Heterocyclic A*β* aggregation inhibitors, polyphenol-derived analogues, and peptide/polyphenol or *β*-sheet-breaker modulators demonstrate that aromatic surface engagement, hydrogen-bonding patterns, lipophilicity, and topology/interface-directed disruption can be systematically optimized [93,94,95,96]. These examples support the proposed design principles of gallate bioisosteric replacement, topology control, and node/interface targeting for chemically stable next-generation modulators.

### 6.1. Bioisosteric Replacement of the Gallate Interaction Motif

The gallate moiety of EGCG plays a central role in mediating multivalent interactions with A*β*, including hydrogen bonding and cation–*π* engagement with residues such as K28, which contribute to the stabilization of *β*-sheet networks in protofibrillar assemblies [23]. However, the ester linkage and polyphenolic character of the gallate group render it metabolically labile and chemically unstable [63]. Accordingly, next-generation scaffolds should incorporate biostable bioisosteric motifs capable of reproducing the electronic and geometric features of the gallate group, specifically, high *π*–electron density and hydrogen-bonding capacity, while improving resistance to oxidative degradation and enzymatic hydrolysis.

Conceptual examples of gallate bioisosteric replacement include heteroaromatic scaffolds, such as quinoline-, isoquinoline-, or benzoxazole-derived motifs, as well as stabilized aromatic carboxamide or carboxylic acid bioisosteric motifs [97,98]. These replacements may be explored to preserve key functional features of the gallate group, including an electron-rich aromatic surface, directional hydrogen-bonding capacity, and potential cation–*π* or *π*–*π* engagement, while improving hydrolytic or metabolic stability. The objective is therefore functional bioisosterism rather than direct structural replication of the catechin ester architecture.

### 6.2. Enforcement of Seeding-Incompetent Contact Topologies

A key mechanistic insight from the experimental studies is that effective inhibition of A*β* toxicity does not necessarily require complete aggregate dissociation, but rather the stabilization of off-pathway, non-templating assemblies. NMR/DEST measurements have demonstrated that EGCG suppresses seeding competence by shifting the *β*-region interactions from direct, templating contacts to more weakly associated tethered configurations [12]. From a design perspective, small molecules should therefore be optimized to bias interpeptide contact networks toward non-productive topologies, reducing the probability of secondary nucleation and fibril propagation. This principle emphasizes the modulation of contact geometry and connectivity, rather than the maximization of binding affinity alone.

### 6.3. Targeting Interfacial Nodes and Structural Vulnerabilities

Amyloid assemblies are stabilized by a network of interfacial interactions, including salt bridges, hydrogen bonds, and hydrophobic packing motifs, which are unevenly distributed across the aggregate structure. Molecular dynamics simulations indicate that EGCG preferentially perturbs structurally labile regions, such as interfacial grooves, *β*-sheet edges, and salt bridge nodes (e.g., D23–K28), leading to local destabilization of the *β*-network [23,25]. Rational design should therefore prioritize interfacial residence and node targeting, enabling selective disruption of aggregation-critical interactions without requiring complete structural disassembly. In this context, shape- and ensemble-based design approaches can be employed to identify scaffolds capable of engaging dynamic interfacial pockets and fluctuating structural motifs.

### 6.4. Integration into Multi-Target-Directed Ligand (MTDL) Frameworks

Given the multi-factorial nature of Alzheimer’s disease, therapeutic efficacy is unlikely to be achieved through modulation of A*β* aggregation alone. Next-generation modulators should therefore be embedded within MTDL architectures that simultaneously address complementary pathological processes, including oxidative stress, metal ion dyshomeostasis, and neuroinflammation. From a medicinal chemistry standpoint, this requires the incorporation of functional groups that enable metal coordination, redox modulation, or enzyme interaction, while preserving the interaction geometries necessary for aggregation modulation. Transitioning from polyphenolic scaffolds to more drug-like heterocyclic systems offers a pathway to achieving this balance, improving pharmacokinetic properties while maintaining mechanistic activity.

### 6.5. Design Implications

Collectively, these principles redefine A*β* modulation as a problem of network-level perturbation rather than as a single-site inhibition. EGCG serves not as a lead compound, but as a mechanistic template, illustrating how polyvalent, state-dependent interactions can be harnessed to destabilize aggregation pathways. Future small molecules should therefore be evaluated based on their ability to reshape conformational ensembles, disrupt seeding-competent architectures, and target aggregation-relevant interfaces, rather than solely on binding affinity or classical structure–activity relationships.

## 7. Future Directions

Enhanced sampling (REMD; metadynamics) is already being applied to quantify EGCG effects on early-stage heteroaggregation (A*β*-hIAPP) via free energy barriers and reduced *β*-sheet formation [43,45]. Future studies should prioritize state-dependent interface residence and contact–topology metrics to connect binding behavior to seeding suppression and *β*-network fragility [12,22,23].

Looking forward, future workflows should extend beyond conventional enhanced sampling toward multi-scale frameworks that integrate atomistic MD, coarse-grained aggregation landscapes, and experimentally constrained ensemble refinement [60,99]. Machine learning-assisted force field refinement for intrinsically disordered proteins may further improve the balance between sampling efficiency and physical realism, particularly for long-timescale oligomer remodeling and polymorph transitions [100,101,102]. Equally important is closed-loop validation, in which NMR, DEST, cryo-EM, FRET/SAXS, and spectroscopic measurements iteratively constrain simulation-derived hypotheses, allowing for mechanistic models, such as topology switching and interface residence bias, to be tested under experimentally falsifiable conditions rather than inferred solely from computational trajectories [52,103,104].

## 8. Conclusions

This review proposes that EGCG modulates A*β* aggregation through a state-dependent mechanism in which its polyvalent chemistry produces distinct effects across monomeric, oligomeric, and protofibrillar assemblies. In monomeric ensembles, EGCG primarily redistributes conformational populations and masks aggregation-prone motifs. In oligomeric assemblies, it supports remodeling toward seeding-incompetent contact topologies; while, in protofibrillar states, it preferentially perturbs interfacial *β*-sheet networks, salt bridge nodes, and hydrogen-bonding stabilizers.

The central mechanistic contribution of this review is the integration of experimental and molecular-simulation evidence into a unified framework that distinguishes experimentally constrained mechanisms, high-confidence MD-supported observations, and model-dependent hypotheses. This distinction is essential for avoiding an overinterpretation of residue-specific binding sites or polymorph-restricted effects while preserving the mechanistic value of reproducible network-level trends.

From a medicinal chemistry perspective, EGCG is best regarded as a mechanistic probe rather than as a direct therapeutic lead. Its value lies in revealing design principles for next-generation amyloid modulators, including state-biased interface engagement, control of seeding-competent contact topology, selective perturbation of *β*-network vulnerabilities, and replacement of chemically labile polyphenolic motifs with more stable drug-like scaffolds. These principles may extend beyond EGCG and provide a broader conceptual basis for designing small-molecule modulators of amyloid aggregation.

### Original Mechanistic MD Analysis: Interface Residence Bias as a State-Discriminating Framework

Building on the framework introduced in this review, IRB is proposed as a qualitative, state-discriminating model for interpreting EGCG–A*β* interactions. In monomeric ensembles, IRB is expected to be weak and distributed because EGCG engages multiple transient pockets across a broad conformational space. In oligomeric assemblies, IRB becomes more functionally focused, reflecting EGCG-induced remodeling of the *β*-region contacts toward growth-incompetent configurations. In protofibrillar assemblies, IRB is strongest because EGCG preferentially engages structurally vulnerable interfacial nodes, including salt bridge and hydrogen-bonding networks that stabilize *β*-sheet architecture.

This framework supports a qualitative ordering of IRB across aggregation states: protofibril > oligomer > monomer. By linking binding localization to mechanistic efficacy, IRB reconciles the apparent discrepancy between diffuse monomeric multi-pocket binding and more localized protofibril network destabilization. This model should be regarded as a conceptual hypothesis that requires further experimental and computational validation, particularly through contact map analysis, topology-specific order parameters, and a closed-loop comparison with structural and biophysical data.

## Figures and Tables

**Table 1:** Key MD Metrics and Mechanistic Readouts Used in EGCG–A*β* Studies.

Metric	Mechanistic Meaning	State Where Most Informative	Representative Sources
RMSD/Rg/SASA	Global destabilization/expansion; solvent exposure changes	Protofibril/fibril; oligomers; monomers	[6,20,23,25,40,41]
*β*-sheet content + interchain H-bonds	*β*-network integrity; templating competence	Oligomers; protofibrils	[4,23,40,41]
Salt bridge persistence (e.g., D23–K28/K28-associated)	Node stability of *β*-architecture	Protofibrils	[23,25]
Contact maps/topology descriptors	Direct vs. tethered growth contacts; seeding competence	Oligomer remodeling	[5,12]

**Table 2:** EGCG–A*β* Interaction Profile (State-Dependent).

Interaction Type	Primary Mechanistic Role	State Emphasis	Representative Sources
H-bond networks	Competes with interchain *β* H-bonds; rewires interfaces	Protofibril/fibril	[23,41,42]
*π*–*π*/CH–*π*	Engages aromatic /hydrophobic motifs; disrupts packing	Protofibril; oligomers	[23,25]
Cation–*π* (gallate-enabled)	Perturbs K28-associated stabilizers/salt bridges	Protofibril	[23]
Multi-pocket occupancy	Redistributes ensembles; masks hot spots	Monomer	[20,24]

## Data Availability

No new data were generated in this study. All data analyzed and discussed are derived from previously published studies and are appropriately cited within the manuscript.

## Supplementary Materials

The following supporting information can be downloaded at: https://www.mdpi.com/article/10.3390/biom16050734/s1, Table S1. Representative structural model sources used in EGCG-A*β* simulation studies [4,12,20,23,25,30,31,40,55,56,57].

## Abbreviations

The following abbreviations are used in this manuscript:

A*β*	Amyloid-beta
A*β*(1–40)	Amyloid-*β* residues 1–40
A*β*(1–42)	Amyloid-*β* residues 1–42
AD	Alzheimer’s disease
APP	Amyloid precursor protein
APP/PS1	APP/presenilin 1
hIAPP	Human islet amyloid polypeptide
EGCG	(-)-Epigallocatechin-3-gallate
EGC	Epigallocatechin
GCG	Gallocatechin gallate
CG	Catechin gallate
CSF	Cerebrospinal fluid
MD	Molecular dynamics
REMD	Replica exchange molecular dynamics
REST2	Replica exchange with solute tempering 2
RMSD	Root mean square deviation
RMSF	Root mean square fluctuation
Rg	Radius of gyration
SASA	Solvent accessible surface area
NMR	Nuclear magnetic resonance
DEST	Dark state exchange saturation transfer
FRET	Förster resonance energy transfer
IRB	Interface residence bias
SAR	Structure–activity relationship
BBB	Blood–brain barrier
MTDL	Multi-target-directed ligand
PAINS	Pan-assay interference compounds
