# Supplementary material for: Epigallocatechin Gallate as a State-Dependent Modulator of Amyloid-β: Molecular Simulation-Guided Mechanistic Synthesis for Structure-Based Inhibitor Design
Source: Biomolecules. 2026 May 17;16(5):734. doi: 10.3390/biom16050734 (PMC13204674; doi:10.3390/biom16050734)
Supplement: Supplementary file 1 [file biomolecules-16-00734-s001.zip › biomolecules-4273375-supplementary.pdf]

biomolecules

MDPI

Supplementary Material

Review

Epigallocatechin Gallate as a State-Dependent Modulator of Amyloid-*β*: Molecular-Simulation-Guided Mechanistic Synthesis for Structure-Based Inhibitor Design

Budimir S. Ilić

Department of Chemistry, Faculty of Medicine, University of Niš, 18000 Niš, Serbia;
budimir.ilic@medfak.ni.ac.rs

Table S1. Representative structural model sources used in EGCG-A*β* simulation studies.

Table S1. Representative structural model sources used in EGCG-Aβ simulation studies.

Aggregation state/model class	Structural basis	Relevance for EGCG-Aβ interpretation	Representative model source
Aβ monomer ensembles	REMD and MD/NMR-derived conformational ensembles of Aβ monomers	Supports interpretation of monomeric Aβ as a heterogeneous ensemble rather than a single binding-competent structure; relevant to multi-pocket binding, hotspot masking, and ensemble redistribution	[4,20,55]
Aβ(1-42) dimer model with EGCG	Atomistic REMD simulations of Aβ(1-42) dimers in the absence and presence of EGCG	Supports EGCG-induced expansion of Aβ dimers, reduced interchain contacts, reduced β-sheet content, and redistribution toward less fibril-prone states	[40]
EGCG-remodeled Aβ oligomers	NMR/DEST-constrained experimental analysis of EGCG-induced remodeling of Aβ oligomers	Supports EGCG-induced conversion of toxic oligomers into seeding-incompetent assemblies, including direct-to-tethered contact-topology switching	[12]
LS-shaped Aβ(1-42) fibril/protofibril model	Cryo-EM-derived LS-shaped Aβ(1-42) fibril structure	Provides a structural template for LS-shaped protofibril/fibril disruption simulations; relevant to groove/ridge asymmetry, fibril-end accessibility, and K28-associated stabilizing contacts	[56]
EGCG/EGC-disrupted LS-shaped Aβ(1-42) protofibril	Microsecond all-atom MD simulations based on the cryo-EM-resolved LS-shaped Aβ(1-42) protofibril/fibril model	Supports gallate-enabled cation-π interaction with K28, disruption of K28-associated salt-bridge contacts, and stronger protofibril-disruptive capacity of EGCG relative to EGC	[23]
EGCG/apigenin-disrupted Aβ(1-42) protofibril	All-atom MD simulations of ligand-Aβ(1-42) protofibril complexes	Supports ligand-induced reduction of β-sheet content, hydrogen-bond disruption, D23-K28 salt-bridge perturbation, and protofibril destabilization	[25]
General Aβ aggregate polymorphism	ssNMR and cryo-EM structural framework for Aβ fibrils and aggregate polymorphism	Supports the methodological point that model choice and polymorph selection condition mechanistic conclusions in Aβ simulations	[30,31,56,57]
